# Communicating science: a responsibility on par with scientific practice

**DOI:** 10.5935/0004-2749.2024-1017

**Published:** 2025-06-24

**Authors:** Marcony R. Santhiago

**Affiliations:** 1 Faculdade de Medicina, Universidade de São Paulo, São Paulo, SP, Brazil

*“What you see is all there is.” - Daniel Kahneman*

If communicating science were an Olympic sport, many scientists would still be warming up on the sidelines, watching international competitors perform their verbal acrobatics with impressive skills. This highlights a pivotal challenge: Many scientists struggle to effectively communicate their research. *Scientific communication* is a fundamental responsibility that transcends the mere disclosure of findings. It is essential for transforming scientific knowledge into practices, public engagement, and social development. In today’s world, in which misinformation and dwindling funding are widespread, communicating science is as vital as conducting it. This editorial explores the importance of scientific communication, analyzes the production of scientific publications in Brazil compared with the United States and China (currently leading countries in terms of the number of publications), and discusses the importance of English language in knowledge dissemination. Furthermore, it emphasizes the efforts of the *Arquivos Brasileiros de Oftalmologia* (ABO) in improving scientific communication within its field and the value of congress presentations as a vital communication tool. This multifaceted approach is critical for maximizing the impact of scientific research.

## Importance of scientific communication

Effective scientific communication is necessary in raising public cognition regarding the importance of research. This heightened awareness is a byproduct and a powerful tool in the fight against misinformation. By sharing their findings, scientists inform society and promote scientific education, which is crucial for the development of a well-informed population. Consequently, scientific communication becomes vital for ensuring that political and social decisions are based on evidence, extending its impact beyond the scientific community.

## The role of the Declaration of Helsinki

The importance of scientific communication is further reinforced by ethical principles such as those established in the Declaration of Helsinki^(1)^, formulated by the World Medical Association. This declaration highlights that research must be conducted with transparency and responsibility and that the results be widely shared to benefit society. Access to scientific information is fundamental, and communication must ensure the integrity and reliability of the presented data, always respecting the rights of the participants involved in the research. Therefore, communicating science advances the pursuit of knowledge and fosters an ethical and responsible environment within scientific research. This ethical framework underscores the responsibility inherent in scientific communication.

## Statistical data: scientific production in Brazil

In 2022, Brazil published approximately 42,000 scientific articles in indexed journals. While this number represents growth over the previous years, the São Paulo Research Foundation (FAPESP)^(2)^ noted a slowdown in production in some areas of science. This observation indicates the need for a broader perspective on scientific progress. Contrarily, the United States leads the global scientific production with approximately 569,000 publications, whereas China, with an explosive growth of 20% per year, reached approximately 557,000 pu-blications^(3^,^4)^.

With an estimated 8,000 to 12,000 articles published in 2022, representing 24% to 29% of the total publications in Brazil^(5)^, the *Universidade de São Paulo* (USP) demonstrates a significant contribution to Brazilian scientific production. However, these numbers are not fully reflected in the global impact of Brazilian research. Strategic actions to increase the visibility and international impact of research are necessary to achieve the full potential of national production.

## What our Arquivos Brasileiros de Oftalmologia has done

The *Arquivos Brasileiros de Oftalmologia*, under the leadership of its editor-in-chief, Newton Kara-Junior, has introduced new tools for improving communication quality and speed. ABO continuously publishes 60 articles per year, always in English, and uses a fast-track system for unpublished articles. These articles are carefully selected for their conceptually relevant contributions. The time-sensitive publication process of the ABO, combined with its presence on social media, expands its reach and amplifies its impact. This demonstrates a commitment to ensuring the timely and accessible dissemination of vital ophthalmological research. This proactive approach serves as a model for other publications^(6^,^7)^.

## The role of languages in scientific communication

Communication in English has become an undeniable necessity in the global academic environment. Majority of scientific publications are available in this language, which ensures broader access to discoveries. Studies have shown that articles published in English often have a higher citation rate. Therefore, proficiency in English scientific communication is not just a beneficial skill but also an integral step toward increasing the visibility and impact of Brazilian science on the international stage. It is a skill that every scientist should strive to master to ensure that their research reaches a broader audience and makes a considerable impact. This emphasis on language proficiency is a powerful motivator for scientists to enhance their communication skills and strive for global scientific recognition^(7)^.

## The changing landscape and its role in communication

There is a socioeconomic context to getting your article published. The current scientific publishing system poses significant challenges, particularly for Brazilian researchers. While high-impact factor journals are often prioritized for continued funding, this system creates barriers for those needing broad institutional support. High publication and reader access fees limit accessibility, making it difficult to effectively communicate the research findings to a wider audience. Such a limited reach directly undermines the impact of scientific work and can disproportionately affect researchers from institutions having fewer resources or from countries whose currencies are less valued internationally compared with publication fees, typically in dollars^(6^,^7)^.

However, we often say that publishing an article is priceless, and could be, I mean, literally.

This context highlights the increasing importance of “diamond journals”-open-access publications that do not charge authors or readers. These journals are gaining recognition for their high-quality content and rising impact factors. Diamond journals generally prioritize clear communication strategies, recognizing that broad accessibility and effective presentation substantially increase the impact of research. The ABO exemplifies this model by combining rigorous research with proactive, multiplatform communication. Their time-sensitive publishing process guarantees the timely and widespread dissemination of key ophthalmological research. The rise of diamond journals and innovative approaches, such as that of the ABO, indicates the urgent need for alternative models that prioritize effective communication, ensuring greater equity and broader impact within scientific publishing. This need for change is pressing and cannot be disregarded^(6^-^8)^.

## Challenges of scientific communication in Brazil

Despite the volume of publications, the actual impact of Brazilian research remains a challenge. Studies using the Web of Science^(4)^ suggest that the average number of citations for Brazilian articles tends to be lower than that of US articles, particularly in high-impact journals. Such a difference can be attributed to several factors, including the publication language (most high-impact journals publish in English), the visibility of Brazilian journals, and open access to articles. To increase the visibility and impact of Brazilian research, strategies such as ABO’s new publication tools, increasing international collaborations, and strengthening scientific dissemination in English are imperative. It is paramount to overcome these obstacles to elevate Brazil’s scientific influence globally.

## The value of congress presentations: posters and oral communications

While publications in peer-reviewed journals remain the gold standard for scientific finding dissemination, the importance of congress presentations-posters and oral-should not be underestimated. These alternative communication avenues offer a unique opportunity to engage a broader audience, often including researchers from diverse backgrounds and career stages. The interactive nature of the congress presentations fosters a direct dialogue, facilitating the immediate exchange of ideas and feedback. This dynamic interaction can stimulate further research and collaborations, potentially leading to more impactful publications.

Furthermore, congresses provide an accessible platform for researchers from institutions with fewer resources to share their findings and gain valuable exposure. Therefore, encouraging participation in congresses as a complementary communication strategy is important for broadening the reach and impact of scientific research. It is a vital element in fostering scientific discourse and collaboration.

## Future predictions and trends

Current predictions indicate that China is likely to surpass the United States in terms of the number of scientific publications in the near future, considering its ongoing growth in research and development investment. However, Brazil is unlikely to participate in this competition at this moment. Despite its history of growth in scientific production, Brazil faces the challenge of reversing the slowdown and securing adequate funding for research projects. This situation requires a joint effort from governments, institutions, and scientists to prioritize communication and international collaboration. By fostering partnerships and sharing knowledge across borders, we can collectively enhance the impact of scientific communication and contribute to the global advancement of science. This collaborative approach is fundamental for future progress.

Scientific communication is not merely a consequence of research but an important element of the entire scientific practice. As we navigate a world filled with information, the effectiveness of communicating science shapes public perception and understanding. The strengthening of communication in science, coupled with the increase in the impact of Brazilian publications and active participation in congress presentations, can be a turning point for developing national research. In a landscape where perception often dictates reality, scientists must focus not only on their discoveries but also on how they convey their findings to the world. Prioritizing communication ensures that scientific knowledge becomes accessible, impactful, and effectively utilized to promote social well-being. Only then can we build a future where the wonders of science truly resonate with and serve society at large. The time for action is now.
